# The Impact of Androgenic Alopecia on the Quality of Life of Male Individuals: A Cross-Sectional Study

**DOI:** 10.7759/cureus.47760

**Published:** 2023-10-26

**Authors:** Omar A Al Najjar, Mohammed A Alkhars, Saleh F Al Molhim, Mohammed S AlAjmi, Abdullah A Alhafith, Maryam A Al Najjar, Abdullah AlMaqhawi

**Affiliations:** 1 Department of Family and Community Medicine, King Faisal University, Al-Ahsa, SAU

**Keywords:** hair loss, self-esteem, psychological impact, male pattern hair loss, kingdom of saudi arabia (ksa), quality of life (qol), male patient, androgenic alopecia

## Abstract

Background

Hair plays a significant role in physical appearance and hair loss can profoundly affect self-esteem and mental health. Studies show that people with clinically obvious and undetectable hair loss may have dramatically decreased quality of life (QoL). This study investigated the impact of androgenic alopecia on the quality of life of male individuals in the Eastern Province of Saudi Arabia and their willingness to seek treatment.

Methods

In the eastern province of Saudi Arabia, a cross-sectional study was carried out among men identified with androgenic alopecia (AGA). A self-administered survey was disseminated among the patients through social media sites. The questionnaire includes fundamental demographic factors including age, place of residence, level of education, the severity of androgenic alopecia, treatment method, and Skindex-29 to assess the patient's quality of life.

Results

Four hundred-two male patients out of 717 participants were selected, and 158 (39.3%) were aged between 20 to 29 years old. Satisfaction with treatment medication was reported by 24 (19.5%) out of those who underwent treatment (n=123). Less effectiveness was the most common reason for treatment dissatisfaction (81, 81.8%). The overall mean Skindex-29 score was 23.2 (SD 19.6) out of 100 points. Younger age, suffering hair loss for a shorter duration, undergoing alopecia treatment, being diagnosed with alopecia by a medical doctor, and having a moderate level of AGA were the factors that greatly affected the patient's QoL.

Conclusion

Consistent with the literature, this study showed that AGA significantly impaired patients' QoL. Among QoL domains, the symptoms domain had a greater effect on patients than the emotions or functional domains. Younger males who were suffering recently from hair loss and were diagnosed with AGA by the medical doctor demonstrated greater QoL impairment than the rest of the patients. A multicenter study may result in a better representation of the impact of QoL in patients with AGA.

## Introduction

Hair plays a significant role in physical appearance and hair loss can have a profound effect on self-esteem and mental health [1]. Studies show that people with clinically obvious and undetectable hair loss may have dramatically decreased quality of life (QoL) [2]. Alopecia is a common condition affecting both genders around the world [3,4]. In their lifetime, around half of men and one-third of women will experience alopecia [5]. Alopecia can be classified into two main categories: scarring and non-scarring. Non-scarring alopecia is more common; it is characterized by progressive hair loss. Androgenic alopecia (AGA) is one category of non-scarring alopecia, and it is considered to be the most common type of hair loss around the world [6]. AGA has two types: male-pattern androgenic alopecia and female-pattern androgenic alopecia, with male-pattern being more prevalent [7].

Although there are several contributing factors to AGA, genetic predisposition and hormonal status particularly play an important role in its etiology [8]. It is believed that male-pattern AGA occurs due to the effects of androgen hormones on hair follicles, and it is characterized by a receding hairline and thinning of hair on the frontal area and the crown of the head because of the high levels of androgen receptors and alpha‐reductase type I and II activities in these areas [9]. The highest prevalence of AGA is in Caucasians with rates around 30-50% in men in their 30s-50s [10]. A study conducted in Jazan, Saudi Arabia, showed that 64% of male participants believe their alopecia is caused by genetic factors [11].

Males with AGA frequently believe that their alopecia is a serious illness that will undoubtedly have a negative effect on their lives and psychosocial side effects [10-12]. Alopecia patients' QoL tends to be greatly affected, including their self-esteem, confidence, relationships, and jobs. A cross-sectional study conducted on 120 patients concluded that AGA had a significant psychological influence on people's thoughts, feelings, and how society interacted with their concerns [13]. Moreover, patients with AGA may have reduced body image satisfaction and fear of social rejection [14]. A study conducted in Poland showed that 60% of Polish men felt ashamed of their baldness, 81.3% of them faced stress in daily life, and 66.7% of them had a significant negative influence on their self-esteem [15]. Since there is a lack of data on the impact of AGA on QoL in Saudi Arabia, the objectives of this study are to assess the impact of androgenic alopecia on the quality of life of male individuals in Saudi Arabia and their willingness to seek treatment for the condition.

## Materials and methods

This is a cross-sectional observational study conducted from April to June 2023 in the Eastern Province of Saudi Arabia, which includes the following areas: Al-Ahsa, Dammam, Al-Khobar, Dhahran, Al-Jubail, Ras Tanurah and Al-Qatif. Saudi male individuals aged between 20 and 60 years who live in these areas, suffer from androgenic alopecia, and consented to participate in the study were included. Subjects aged less than 20 or more than 60, female gender, and those who live outside the eastern province were excluded. The validity of the questionnaire was tested by distributing it to a small group of participants. Based on the results, a few changes were made to the questionnaire, which then was circulated on a larger social media platform. An online questionnaire was created in Arabic language using Google Forms and distributed on Social Media platforms (Twitter, Telegram, and WhatsApp), which involved the consent request before study participation. Ethical clearance was taken from the Institutional Review Board (IRB) at King Faisal University, Reg No KFU-REC-2023-APR-ETHICS785.

Demographic data

Participants were asked to complete a questionnaire regarding their demographic information, including their gender, age, education level, disease duration, history of treatment, and level of satisfaction with the care they received (see Appendix A). The participants were divided into three groups based on their age: those between 20 and 30, those between 31 and 50, and those between 51 and 60 to compare the participant QoL with respect to various age features. In addition, to compare the participant QoL across different educational levels, the participants were separated into four groups: elementary or lower, high school, diploma, and bachelor. The participants were divided into four groups to compare the duration of the disease and the participants' QoL: less than five years of AGA, five years of AGA, five to 10 years of AGA, and over 10 years of AGA. Participants were divided into groups with high and low levels of prior treatment satisfaction to compare the patient's quality of life in light of varied levels of treatment satisfaction.

Severity of androgenic alopecia

The severity of AGA was evaluated using the Hamilton-Norwood visual scale (see Appendix B). A visual diagram of the scale was uploaded to the questionnaire so the participants could choose the degree of their AGA by choosing the appropriate shape of their hair from the diagram. There are seven groups that make up the usual pattern of hair loss. 'Type I' refers to the absence of hair loss. 'Type II' refers to frontal hairline regression that is just mild. Type III signifies even more frontal loss and is regarded as "cosmetically significant." The subgroup of type III, known as "III vertex," has severe frontal recession as well as hair loss in the scalp's vertex area. The frontal and vertex continue to lose hair in types IV through VI, while type VII is the last to retain a considerable volume of hair on the scalp. The degree of hair loss is classified into three categories. The H-N scale categories are mild (Type I, II), moderate (Type III, III Vertex, and IV), and severe (Type V, VI, and VII) [16].

Evaluation of quality of life

For evaluating the QoL, a self‐reported dermatology-specific QoL measurement tool, Skindex-29, which was initially developed by Chren MM [17], was modified to assess the QoL of patients with AGA to be applicable for those patients with alopecia by replacing the word "skin" with "scalp" and "skin condition" by "alopecia" will be used [18]. Permission from Mapi Research Trust for translating the scale into Arabic version was taken. Skindex-29 scale questionnaire composed of 29 items, investigating three domains: symptoms (7 items), functional impact (12 items), and effect on emotions (10 items). Participants responded to each question with a score ranging from 1 (never bothered) to 5. (Always bothered). Each response was converted to a linear score between 0 (never bothered) and 100. (Always bothered). A high score indicates severely impaired QoL and a low score reflects mild damage in the QoL.

Statistical analysis

The data were computed using the software program Statistical Packages for Software Sciences (SPSS) version 26.0 (IBM Corp., Armonk, NY). Categorical variables were elaborated as frequencies and proportions (%). Continuous variables were shown as mean, standard deviation, and median (min-max). The scores of Skindex and its domains were compared with socio-demographic characteristics and related alopecia conditions of the male patients by using the Mann Whitney Z-test and Kruskal Wallis H-test. Statistical collinearity was measured using the Shapiro-Wilk test as well as the Kolmogorov-Smirnov test. All scores of Skindex and its domains follow the non-normal distribution. Thus, the non-parametric tests were applied between comparisons. Values were considered significant with a p-value of less than 0.05, and values of less than 0.001 were considered highly statistically significant.

## Results

Four hundred and two male patients were enrolled. As seen in Table 1, 158 (39.3%) were aged between 20 to 29 years old. More than half (214, 53.2%) lived in Al-Ahsa, and 243 (60.4%) were bachelor's degree holders.

**Table 1:** Socio-demographic characteristics of male patients (n=402) Values are presented as numbers and percentages (%).

Study data		N (%)
Age group		
· 20 – 29 years	158 (39.3%)	
· 30 – 39 years	111 (27.6%)	
· 40 – 49 years	70 (17.4%)	
· 50 – 60 years	63 (15.7%)	
Living area		
· Al-Ahsa	214 (53.2%)	
· Dammam	66 (16.4%)	
· Al-Khobar	51 (12.7%)	
· Dhahran	22 (05.5%)	
· Al-Jubail	42 (10.4%)	
· Ras Tanurah	01 (0.20%)	
· Al-Qatif	06 (01.5%)	
Educational level		
· Elementary or below	08 (02.0%)	
· High school		67 (16.7%)
· Diploma		84 (20.9%)
· Bachelor		243 (60.4%)

Table 2 shows that 135 (33.6%) of the patients had a duration of alopecia of less than five years. The prevalence of patients who underwent hair loss treatment was 123 (30.6%), and the most commonly mentioned treatment method was medical treatment (61, 49.6%). Among those who underwent treatment (n=123), only 24 (19.5%) indicated satisfaction. Among those who were dissatisfied with the treatment (n=99), the most common reason was less effective (81, 81.8%). Regarding the treatment options that were undertaken in this study, 123 (30.6%) underwent hair treatment, mainly by medical treatment, e.g., minoxidil, finasteride (61, 49.6%), hair shampoo (57, 46.3%), herbal therapy (33, 26.8%), hair transplant (23, 18.7%), some patients applied light therapy (9, 7.3%) and wigs or hairpieces (7, 5.7%). Despite various treatment methods, patients' satisfaction with treatment was very low (24, 19.5%). Dissatisfaction with treatment was primarily related to the ineffectiveness of the treatment (81, 81.8%), followed by long-term treatment (42, 42.4%) and high cost (33, 33.3%). The prevalence of patients diagnosed with androgenic alopecia by a medical doctor was 193 (48%) and the most common type of androgenic alopecia was type III (59, 14.7%).

**Table 2:** Alopecia and hair condition (n=402) Values are presented as numbers and percentages (%). †Variable with multiple response answers.

Variables	N (%)
Duration from suffering hair loss	
· <5 years	135 (33.6%)
· 5 years	72 (17.9%)
· 6 – 10 years	110 (27.4%)
· >10 years	85 (21.1%)
Has this condition been treated before	
· Yes	123 (30.6%)
· No	279 (69.4%)
Type of treatment ^(n=123) †^	
· Medical treatment (Minoxidil, Finasteride)	61 (49.6%)
· Hair shampoo	57 (46.3%)
· Herbal therapy	33 (26.8%)
· Hair transplant	23 (18.7%)
· Plasma injection	23 (18.7%)
· Light therapy	09 (07.3%)
· Wigs or hairpieces	07 (05.7%)
· Others	12 (09.8%)
Were you satisfied with the medication? ^(n=123)^	
· Yes	24 (19.5%)
· No	99 (80.5%)
Reason for dissatisfaction with medication ^(n=99) †^	
· Low effects	81 (81.8%)
· High cost	33 (33.3%)
· Required long-term treatment	42 (42.4%)
· Others	05 (05.1%)
Were you diagnosed before by a medical doctor with Androgenic Alopecia?	
· Yes	193 (48.0%)
· No	209 (52.0%)
Assessment of androgenic alopecia	
· I	59 (14.7%)
· II	44 (10.9%)
· III	52 (12.9%)
· III Vertex	59 (14.7%)
· IV	33 (08.2%)
· V	24 (06.0%)
· VI	45 (11.2%)
· VII	59 (14.7%)
· Others	27 (06.7%)

In Table 3, it was observed that the symptoms domain showed the highest mean score (mean: 25.6), followed by the emotion domain (mean: 24.6) and functioning domain (mean: 20.6). The overall mean score of Skindex was 23.2 (SD 19.6).

**Table 3:** Assessment of quality of life using hair specific skindex-29 (n=402) Values are presented as mean ± SD, median, minimum, and maximum.

SKINDEX DOMAINS	MEAN ± SD	MEDIAN	MIN - MAX
Emotions	24.6 ± 19.8	22.5	0.00 – 82.5
Symptoms	25.6 ± 20.8	21.4	0.00 – 85.7
Functioning	20.6 ± 21.1	13.6	0.00 – 90.9
Overall	23.2 ± 19.6	18.7	0.00 – 82.1

When measuring the differences in the scores of Skindex according to the socio-demographic characteristics and related alopecia condition of the patients (Table 4), it was found that the younger age group was more associated with higher scores in the emotion domain (H=45.841; p<0.001), symptoms domain (H=30.210; p<0.001), functioning domain (H=25.560; p<0.001) and overall Skindex (H=36.812; p<0.001). Patients with a shorter duration since diagnosis were more associated with higher scores in the emotion domain (Z=7.817; p<0.001), symptoms domain (Z=6.604; p<0.001), functioning domain (Z=8.229; p<0.001), and overall Skindex (Z=7.986; p<0.001). Also, patients who had undergone treatment were more associated with higher scores in the emotion domain (Z=4.337; p<0.001), symptoms domain (Z=3.795; p<0.001), functioning domain (Z=4.402; p<0.001), and overall Skindex (Z=4.439; p<0.001). The scores were statistically significantly higher in the emotion domain (Z=7.817; p<0.001), symptoms domain (Z=6.604; p<0.001), functioning domain (Z=8.229; p<0.001), and overall Skindex (Z=7.986; p<0.001) among patients who were diagnosed with androgenic alopecia by a medical doctor. Finally, patients with moderate androgenic alopecia were more associated with higher scores in the emotion domain (H=20.646; p<0.001), symptoms domain (H=17.276; p<0.001), functioning domain (H=12.795; p=0.002), and overall Skindex (H=17.187; p<0.001). Based on our results, the overall mean score of QoL was 23.2 (SD 19.6) out of 100 points. Regarding Skindex domains, the symptoms domain showed the highest mean score (mean: 25.6), followed by the emotion domain (mean: 24.6), and the least was the functional domain (mean: 20.6). This indicates that patients were more affected by AGA symptoms, while emotion and function domains had a lesser effect on their QoL. These findings seem to be better than the study of Han et al. [18]. According to reports, the global mean score of Skindex was 27.3 (SD 19.1), and the symptoms subscale rated the highest mean with 32.1, followed by the symptom subscale (mean: 26.3) and the function subscale (mean: 24).

**Table 4:** Differences in the scores of Skindex in relation to the socio-demographic characteristics and related alopecia conditions of the male patients (n=402) Values are presented as mean ± SD; ^a^  p-value has been calculated using the Kruskal Wallis H-test; ​​​​​​​^b^ p-value has been calculated using the Mann Whitney Z-test; ** Significant at p<0.05 level; *** Highly significant at p<0.001 level.

Factor	Emotions Score (100) Mean ± SD	Symptoms Score (100) Mean ± SD	Functioning Score (100) Mean ± SD	Overall Score (100) Mean ± SD
Age group				
20 – 29 years	30.8 ± 19.9	31.5 ± 21.6	25.9 ± 22.6	29.1 ± 20.2
30 – 39 years	26.6 ± 20.2	26.7 ± 21.2	21.3 ± 22.0	24.5 ± 20.4
≥40	15.7 ± 15.8	17.8 ± 16.7	13.6 ± 16.2	15.4 ± 15.3
H-test; p-value ^a^	45.841; <0.001**	30.210; <0.001**	25.560; <0.001**	36.812; <0.001**
Educational level				
Diploma or below	26.4 ± 21.5	27.4 ± 21.4	22.5 ± 22.4	25.1 ± 20.9
Bachelor's degree	23.4 ± 18.6	24.5 ± 20.3	19.3 ± 20.2	22.1 ± 18.7
Z-test; p-value ^b^	1.100; 0.271	1.384; 0.166	0.954; 0.340	1.123; 0.261
Duration from suffering hair loss				
≤5 years	27.9 ± 19.1	29.2 ± 20.5	22.9 ± 21.7	26.3 ± 19.5
>5 years	21.0 ± 19.9	21.8 ± 20.5	18.1 ± 20.3	20.1 ± 19.4
Z-test; p-value ^b^	7.817; <0.001**	6.604; <0.001**	8.229; <0.001**	7.986; <0.001**
Undergone previous treatment				
Yes	30.7 ± 19.7	31.3 ± 21.2	27.6 ± 22.7	29.7 ± 20.2
No	21.9 ± 19.2	23.1 ± 20.1	17.5 ± 19.6	20.5 ± 18.7
Z-test; p-value ^b^	4.337; <0.001**	3.795; <0.001**	4.402; <0.001**	4.439; <0.001**
Diagnosed by a medical doctor				
Yes	35.2 ± 20.6	34.6 ± 20.9	32.6 ± 21.9	34.0 ± 20.3
No	18.8 ± 16.7	32.6 ± 21.9	13.9 ± 17.4	17.3 ± 16.5
Z-test; p-value ^b^	7.817; <0.001**	6.604; <0.001**	8.229; <0.001**	7.986; <0.001**
Severity of androgenic alopecia				
Mild	24.9 ± 17.7	25.6 ± 19.9	18.1 ± 20.3	22.4 ± 18.1
Moderate	29.5 ± 21.3	30.0 ± 21.2	25.6 ± 21.6	28.1 ± 20.7
Severe	18.9 ± 19.4	20.4 ± 20.9	17.8 ± 21.4	18.8 ± 19.8
H-test; p-value ^a^	20.646; <0.001**	17.276; <0.001**	12.795; 0.002 **	17.187; <0.001**

Table 5 shows that in the emotions domain, the mean difference was statistically significant between moderate and severe alopecia (p<0.001). This has been observed in the symptoms domain, where the mean differences between moderate and severe alopecia were statistically significant (p<0.001). In the functioning domain, the mean difference was statistically significant between mild and moderate alopecia (p=0.018) and moderate to severe alopecia (p=0.007). Finally, in the overall Skindex, a significant mean difference was observed between moderate and severe alopecia (p<0.001).

**Table 5:** Multiple mean comparisons for the severity of androgenic alopecia in relation to the scores of Skindex-29 (n=402) Post hoc analysis has been calculated using the Dunn-Bonferroni test. * The mean difference is significant at the p<0.05 level.

Dependent Variable	(I) Severity	(J) Severity	Mean Difference (I-J)	Std. Error	Sig.	95% Confidence Interval	
						Lower Bound	Upper Bound
Emotions	Mild	Moderate	-4.58670	2.54827	0.218	-10.7149	1.5415
		Severe	6.04047	2.61385	0.064	-0.2455	12.3264
	Moderate	Mild	4.58670	2.54827	0.218	-1.5415	10.7149
		Severe	10.62717^*^	2.39881	0.000	4.8584	16.3960
	Severe	Mild	-6.04047	2.61385	0.064	-12.3264	0.2455
		Moderate	-10.62717^*^	2.39881	0.000	-16.3960	-4.8584
Symptoms	Mild	Moderate	-4.42046	2.68432	0.301	-10.8759	2.0349
		Severe	5.19325	2.75339	0.180	-1.4283	11.8148
	Moderate	Mild	4.42046	2.68432	0.301	-2.0349	10.8759
		Severe	9.61372^*^	2.52687	0.000	3.5369	15.6905
	Severe	Mild	-5.19325	2.75339	0.180	-11.8148	1.4283
		Moderate	-9.61372^*^	2.52687	0.000	-15.6905	-3.5369
Functioning	Mild	Moderate	-7.57560^*^	2.73628	0.018	-14.1560	-0.9953
		Severe	0.31581	2.80669	1.000	-6.4339	7.0655
	Moderate	Mild	7.57560^*^	2.73628	0.018	0.9953	14.1560
		Severe	7.89141^*^	2.57579	0.007	1.6970	14.0858
	Severe	Mild	-0.31581	2.80669	1.000	-7.0655	6.4339
		Moderate	-7.89141^*^	2.57579	0.007	-14.0858	-1.6970
Total Skindex score	Mild	Moderate	-5.71935	2.54190	0.075	-11.8322	0.3935
		Severe	3.57969	2.60730	0.512	-2.6905	9.8499
	Moderate	Mild	5.71935	2.54190	0.075	-0.3935	11.8322
		Severe	9.29905^*^	2.39280	0.000	3.5447	15.0534
	Severe	Mild	-3.57969	2.60730	0.512	-9.8499	2.6905
		Moderate	-9.29905^*^	2.39280	0.000	-15.0534	-3.5447

## Discussion

Patients with AGA are greatly influenced by self-image satisfaction, leading to some adverse psychological effects and subsequently affecting the patient's QoL [19]. The present study is conducted to determine the impact of androgenic alopecia on the quality of life of male individuals in Saudi Arabia. In this study, we used Skindex-29 to assess the patient's QoL. In India, reports showed that the age group <30 years was less affected by the symptoms subscale; however, they showed higher scores for stigmatization, functioning, and emotions than the older subjects (age >30 years) [20]. However in Poland, 81.3% of the patients sometimes felt stress with their everyday life, around two-thirds (66.7%) reported being negatively affected by the AGA toward their self-esteem, and 60% felt embarrassed by their baldness, mainly in the younger age group (age 18 to 25 years) [15]. AGA appears to have a significant effect on patients' QoL. Therefore, a psychological evaluation should be considered when treating a patient with AGA.

Data from our study suggest that being younger, suffering from hair loss for a shorter duration (≤5 years), having undergone hair treatment, being diagnosed with AGA by a medical doctor, and having a moderate level of AGA were the significant predictors of increased risk for impaired QoL. Furthermore, multiple comparisons between the severity of AGA according to the QoL and its domains revealed significant variations, particularly between moderate to severe AGA in relation to global score and in all QoL domains, including emotions, symptoms, and functions. In China, health-related QoL (HRQoL) was significantly impaired in younger patients (<30 years), lower education, single patients, and urban residents [21]. However, a study done in Finland found no significant association between AGA diagnosis and its severity in terms of anxiety, depression, quality of life, self-esteem, and sexual symptoms, adding that the severity of AGA showed lower sexual activity when compared with those without AGA, but the results did not reach statistical significance (p>0.05) [8]. Similarly, Reid et al. (2012) documented that clinical assessment of hair loss severity (HLS) did not reliably predict the patient's QoL or the patient's perception of HLS [2]. Incidentally, in a systematic review based on 13 studies, all studies agreed that AGA serves as a significant psychosocial stressor in the lives of those affected as it impairs QoL according to multiple measures [14].

Management and treatment are essential factors for improving QoL. According to the study published in Riyadh, 60% of the patients indicated that vitamins and minerals were the most commonly recommended for at least 1 type of hair loss [5]. In the Jazan Region, there were some variations of beliefs between male and female respondents, wherein males believe that hair loss is hereditary, while females believe it is due to the lack of vitamins and minerals [11]. Surprisingly, 58.3% of the respondents did not seek medical advice for the treatment of the disease, while approximately 25.3% used medications to treat their hair loss. According to the review article conducted by Stough et al. [12], both minoxidil and finasteride can stop hair loss and help to regrow hair at some points; however, neither medication can effectively restore all hair loss or reverse complete baldness. 

A study suggested that treatment for the complete rejuvenation of hair is still not available at present times, but pharmacotherapy can stop or partially reverse hair loss [21]. Albeit our results seem to agree with this scenario. According to Stough et al. [12], satisfaction with the outcome of treatment correlates positively with the level at which objective improvement in hair growth varies noticeably from patient to patient. The author further suggests that appropriate education is vital to inform patients about the possible gaps and disadvantages and the potential promise of different intervention modalities to understand the methods of management used prior to initiation.

This study's limitations include its limited applicability to other forms of alopecia, as well as, its lack of control for participants' concurrent anxiety and/or depression, and the use of an online questionnaire, which raises concerns about the possibility of selection bias and systematic bias.

## Conclusions

Depending on disease severity, the quality of life of male patients is greatly affected by androgenic alopecia. Younger patients diagnosed recently with AGA by the medical doctor were more likely to exhibit significant impairment in quality of life than any other subjects. Some patients tried treatment methods, mainly medications, but few expressed satisfaction due to ineffectiveness. This study showed evidence that AGA is a detrimental factor in male QoL. Hence, it is worth considering psychological evaluation when treating patients diagnosed with AGA. Since almost 80% of our population was dissatisfied with the therapy they attempted, more research on the efficiency of AGA treatment is advised in order to get more accurate findings.

## Figures and Tables

**Figure 1:**
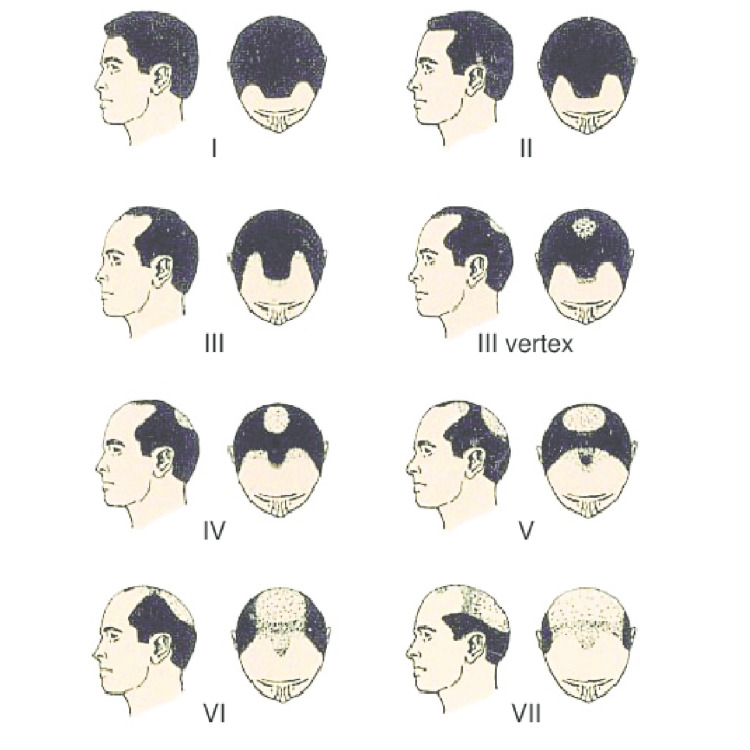
The Norwood–Hamilton scale of male pattern baldness. There are seven groups that make up the usual pattern of hair loss. 'Type I' hair loss refers to the absence of hair loss. 'Type II' refers to frontal hairline regression that is just mild. Type III signifies even more frontal loss and is regarded as "cosmetically significant."  The subgroup of type III known as "III vertex" has severe frontal recession as well as hair loss in the scalp's vertex area. In types IV through VI, the frontal and vertex continue to lose hair, while type VII is the last to retain a considerable volume of hair on the scalp. Reproduced from Norwood, O.T. (1973) Hair Transplant Surgery (1st edition), courtesy of Charles C. Thomas, Publisher, Ltd, Springfield, Illinois, USA (fig001jem).

## Appendices

**Table 6:** Appendix A: Questionnaire used to obtain the data First part: obtaining consent from the participants and whether to include them or not by answering the second question of the first part. Second and third part: Participants will be asked to complete a questionnaire regarding their demographic information, including their gender, age, education level, disease duration, history of treatment, and level of satisfaction with the care they received. Fourth part: The severity of AGA will be evaluated using the Hamilton–Norwood visual scale (Figure 1).

First part
Do you agree to participate in this research
Do you suffer from Androgenic Alopecia
Second part
Living area
Gender
Age
Education
Third part
How long have you been suffering from hair loss
Has this condition been treated before
Were you satisfied with the treatment
If NO, mention why
Were you diagnosed before by a medical doctor with Androgenic Alopecia
Fourth part
Select the proper shape of your hair from the picture (see Appendix B)
